# Implementing Pay-it-Forward STI Testing: A Mixed-Methods Evaluation in Community-Based Organizations and STI Clinics in China

**DOI:** 10.21203/rs.3.rs-10471326/v1

**Published:** 2026-11-01

**Authors:** Lan Li, Thomas S. Fitzpatrick, Edward Li, Zixuan Zhu, Ye Liu, Ke Zhou, Liyuan Zhang, Gifty Marley, Rayner K.J. Tan, Dan Wu, Jiehu Yuan, Peizhen Zhao, Katherine Li, Cheng Wang, Joseph D. Tucker, Rohit Ramaswamy, Weiming Tang

## Abstract

Background Pay-it-forward is an implementation strategy that offers individuals a health service as a gift and then invites voluntary contributions to support subsequent users. Although prior trials have demonstrated its effectiveness in increasing sexually transmitted infection (STI) testing, little is known about pay-it-forward implementation in diverse settings. Methods We conducted a convergent mixed-methods evaluation guided by the RE-AIM framework. Data was collected from the PIONEER trial, which evaluated a pay-it-forward strategy to promote STI testing in two settings in China: community-based organizations (CBOs) and STI clinics. Quantitative data from 800 trial participants (400 CBOs, 400 clinics) assessed reach, uptake, donation behavior, and participant understanding. Implementers (n = 34) were surveyed to assess organizational readiness. Semi-structured interviews were conducted with implementers (n = 10) to explore adoption processes, workflow adaptations, and perceptions of sustainability, as well as with trial participants (n = 24) to understand their experiences with pay-it-forward. Results Test uptake in pay-it-forward arm was high in both settings (CBO: 90.8%; clinic: 97%), though each setting reached distinct populations. CBOs primarily engaged men who have sex with men and first-time testers, while clinics reached more men with multiple concurrent sex partners. Barriers and facilitators to adoption differed by setting. CBOs had strong mission alignment but chronic staffing shortages, while clinics required leadership approval, multi-department coordination, and implementer motivation to sustain delivery. Clinics frequently abbreviated procedures, including providing full explanations of pay-it-forward, due to workflow pressures. Fewer than half of participants (41%) fully understood the pay-it-forward strategy in both settings. Donation rates were modest and did not differ significantly between settings (CBO: 16.8%; clinic: 21.8%; p = 0.088), though CBO donors contributed larger amounts on average (0.8 vs. 0.4 USD); together, donations covered the cost of approximately 22 additional tests. Qualitative data suggested CBO donations were motivated by gratitude and a sense of community reciprocity, while clinic donations, though somewhat more frequent, were tempered by implementer caution around patient trust and fears of appearing to solicit money. Conclusions Pay-it-forward can increase STI testing in both community and clinic settings; however, test receipt through pay-it-forward does not ensure participants will understand the participatory implementation strategy. CBOs can leverage preexisting connections and trust with marginalized communities to implement pay-it-forward, but resource limitations remain a substantial barrier. The large patient volumes in STI clinics pose a challenge to consistent and faithful implementation of pay-it-forward. Effective scale-up will require setting-specific strategies that address staffing constraints in CBOs and leadership and workflow barriers in STI clinics. Our findings may be relevant to implementation scientists working across clinic and community settings.

